# Sustained Release of Salicylic Acid for Halting Peri-Implantitis Progression in Healthy and Hyperglycemic Systemic Conditions: A Gottingen Minipig Model

**DOI:** 10.1021/acsbiomaterials.4c00161

**Published:** 2024-04-09

**Authors:** Edmara T. P. Bergamo, Lukasz Witek, Ilana Santos Ramalho, Adolfo Coelho de Oliveira Lopes, Vasudev Vivekanand Nayak, Andrea Torroni, Blaire V. Slavin, Estevam A. Bonfante, Kathryn E. Uhrich, Dana T. Graves, Paulo G. Coelho

**Affiliations:** † Department of Prosthodontics, NYU Dentistry, New York, New York 10010, United States; ‡ Biomaterials Division, NYU Dentistry, New York, New York 10010, United States; § Department of Biomedical Engineering, NYU Tandon School of Engineering, Brooklyn, New York 11201, United States; ∥ Hansjörg Wyss Department of Plastic Surgery, NYU Grossman School of Medicine, New York, New York 10016, United States; ⊥ Department of Prosthodontics and Periodontology, University of Sao Paulo, Bauru School of Dentistry, Bauru, SP 17012-230, Brazil; # Department of Biochemistry and Molecular Biology, University of Miami Miller School of Medicine, Miami, Florida 33136, United States; ∇ University of Miami Miller School of Medicine, Miami, Florida 33136, United States; ○ Department of Chemistry, University of California Riverside, Riverside, California 92521, United States; ◆ Department of Periodontics, School of Dental Medicine, University of Pennsylvania, Philadelphia, Pennsylvania 19104, United States; ¶ Division of Plastic Surgery, Department of Surgery, University of Miami Miller School of Medicine, Miami, Florida 33136, United States

**Keywords:** dental implants, osseointegration, peri-implantitis, treatment, metabolic diseases

## Abstract

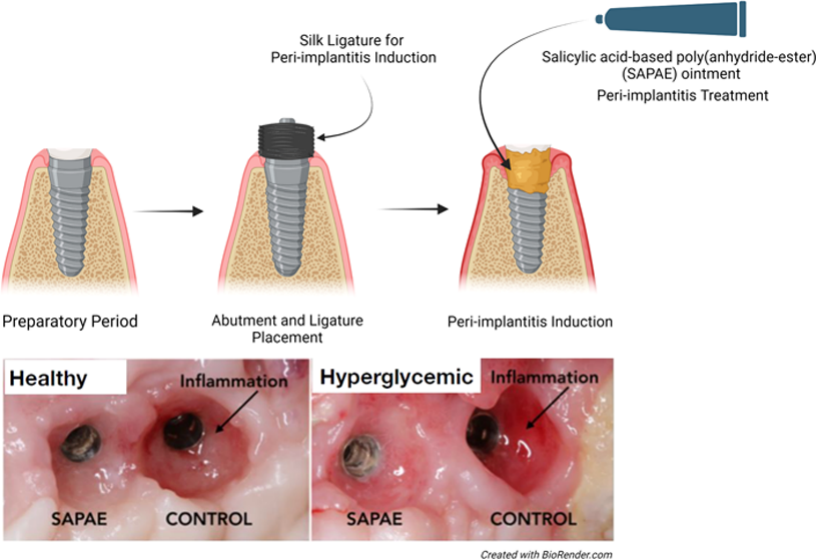

To develop a peri-implantitis model in a Gottingen minipig and evaluate the effect of local application of salicylic acid poly(anhydride-ester) (SAPAE) on peri-implantitis progression in healthy, metabolic syndrome (MS), and type-2 diabetes mellitus (T2DM) subjects. Eighteen animals were allocated to three groups: (i) control, (ii) MS (diet for obesity induction), and (iii) T2DM (diet plus streptozotocin for T2DM induction). Maxillary and mandible premolars and first molar were extracted. After 3 months of healing, four implants per side were placed in both jaws of each animal. After 2 months, peri-implantitis was induced by plaque formation using silk ligatures. SAPAE polymer was mixed with mineral oil (3.75 mg/μL) and topically applied biweekly for up to 60 days to halt peri-implantitis progression. Periodontal probing was used to assess pocket depth over time, followed by histomorphologic analysis of harvested samples. The adopted protocol resulted in the onset of peri-implantitis, with healthy minipigs taking twice as long to reach the same level of probing depth relative to MS and T2DM subjects (∼3.0 mm), irrespective of jaw. In a qualitative analysis, SAPAE therapy revealed decreased levels of inflammation in the normoglycemic, MS, and T2DM groups. SAPAE application around implants significantly reduced the progression of peri-implantitis after ∼15 days of therapy, with ∼30% lower probing depth for all systemic conditions and similar rates of probing depth increase per week between the control and SAPAE groups. MS and T2DM conditions presented a faster progression of the peri-implant pocket depth. SAPAE treatment reduced peri-implantitis progression in healthy, MS, and T2DM groups.

## 1 Introduction

Dental implants represent one of the most important breakthroughs and successful treatment modalities of oral rehabilitation, with approximately a 95% survival rate after 10 years of follow-up.^1^ Nevertheless, inflammation and destruction around implants, such as peri-implant mucositis and peri-implantitis, have increasingly been reported, with a prevalence of approximately 45%,^2−5^ where the severity of tissue breakdown is associated with time in function of the implant.^6,7^ Both peri-implant diseases are bacteria-induced and host-mediated inflammatory processes characterized by considerable tissue damage around the implant.^8^ The watershed sign to distinguish peri-implant mucositis and peri-implantitis lies in the presence of epithelial and connective tissue degradation associated with bone resorption that results in osteolytic lesions.^9,10^ Local and systemic factors, such as history of periodontitis, width of the keratinized tissue, prosthesis overcontouring and impaired oral hygiene, smoking, genetic susceptibility, implant design, and surface characteristics at the transmucosal portion, and systemic conditions, such as pro-inflammatory metabolic diseases, have been identified as risk factors for peri-implantitis onset and/or increased disease severity.^11,12^

Diabetes mellitus (DM) is a chronic metabolic disorder characterized by abnormal carbohydrate, lipid, and protein metabolism and, consequently, persistent hyperglycemia, resulting from deficient insulin secretion and/or action.^13^ There are currently two primary classifications of DM: (i) type 1 (T1DM), which is characterized by an autoimmune destruction of insulin-producing β-cells in the pancreas by pathogenic T cells, influenced by genetic susceptibility and environmental factors, leading to permanent deficiency of insulin; and (ii) type-2 diabetes (T2DM), which is characterized by a decreased responsiveness to insulin combined with insufficient insulin production due to β cells exhaustion resulting from increased insulin secretion needed to maintain normoglycemia.^14^ T2DM development, which represents approximately 90% of individuals with DM,^15^ has been associated with the establishment of an insulin resistance state triggered in a complex pathophysiological scenario by obesity/metabolic syndrome (MS).^16^

Metabolic syndrome (MS) is characterized by a cluster of metabolic disturbances that is diagnosed by the presence of any 3 of the 5 disorders: (i) obesity (elevated waist circumference that is population- and country-specific defined), (ii) drug treatment or elevated triglycerides (≥150 mg/dL to 1.7 mmol/L), (iii) drug treatment or reduced high-density lipoprotein cholesterol (HDL-C) (<40 mg/dL to 1.0 mmol/L in males and <50 mg/dL to 1.3 mmol/L in females), (iv) drug treatment or elevated blood pressure (systolic ≥ 130 and/or diastolic ≥ 85), and (v) drug treatment or elevated fasting glucose (≥110 mg/dL).^17,18^ MS plays a key role in the regulation of glucose levels due to the development of an insulin-resistant state.^16^ Increased abdominal and visceral adipose tissue contributes to insulin resistance, creating a state of chronic hyperinsulinemia as a compensatory mechanism.^19^ Excess free fatty acids (FFAs) produce toxic lipid metabolites and excess glucose leads to high levels of advanced glycation end products (AGEs), both of which cause oxidative stress and are pro-inflammatory.^20,21^ Thus, the pancreatic environment with prolonged insulin resistance leads to the loss of β-cells, intensifying the compromise in the glucose–insulin homeostasis.^22^ Additionally, the expansion of adipose tissue deposits in MS leads to an increased production of pro-inflammatory cytokines along with macrophages and pathogenic T-cell infiltration that create a chronic systemic inflammation.^23,24^ All of the aforementioned effects of MS associated with genetic predisposition are major factors for intensifying the insulin resistance state, leading to T2DM development.^25^

Diverse scientific findings have demonstrated that implant procedures are safe and predictable in patients with well-controlled metabolic diseases, with a survival rate similar to that of healthy patients.^26,27^ Nonetheless, patients with poorly controlled glucose levels have shown lower initial stability and delayed osseointegration and elevated risk of peri-implantitis.^26−29^ Compromised healing around implants has also been reported in a highly translational preclinical model where animals suffering from MS and T2DM, presented with a reduction of approximately 75% in biomechanical and 10–20% in bone formation parameters relative to healthy animals.^30^ Moreover, the prevalence of peri-implant diseases in patients affected by MS and T2DM was almost 2-fold higher compared with healthy patients,^31−33^ with peri-implant crestal bone level exhibiting a proportional relation to glycemic levels.^33^ The factors that enhance peri-implantitis are likely to be related to increased inflammation that predisposes to osteoclastogenesis and reduced bone coupling.^34−38^ Hyperglycemia has also been demonstrated to shift the oral microbiota profile and increase the number of pathogens in salivary and peri-implant sites,^39,40^ as well as affecting their pathogenicity by uncontrolled glycemia and inflammation levels,^34,41,42^ which still needs further investigations.

To date, established peri-implantitis is difficult to treat and tends to progress, ultimately leading to implant failure (e.g., loss). The current methods for the treatment of peri-implantitis have focused on mechanical debridement,^43−47^ local and systemic administration of antimicrobial agents, as well as regenerative procedures with the use of bone graft materials and membranes.^48,49^ The reported efficacy of the different treatment approaches in halting disease progression has been unpredictable or has limited success,^3,50−52^ especially in individuals with pro-inflammatory systemic conditions,^48,53−56^ where the impaired wound healing demonstrated in scenarios of uncontrolled glucose levels is also indicative of a compromised regenerative ability.^30,57−60^ Therefore, the development of therapies that potentially overcome the exacerbated immune-inflammatory response of MS and T2DM, providing a favorable scenario for disease control and bone regeneration around implants, is paramount.

Preclinical tests of efficacy are typically carried out in large pre-clinical animal models that reproduce conditions more accurately than small animal models. This is important in preclinical models that reproduce the oral environment to monitor the pathogenesis and progression of peri-implant diseases as well as the influence of the systemic condition.^61,62^ The current study aimed to develop a highly translational model to test the treatment of peri-implantitis under normal and metabolically compromised conditions such as MS and T2DM. The Gottingen minipig serves this purpose well since anatomy and bone pathophysiology are remarkably similar to humans. The efficacy was evaluated by local application of salicylic acid-based poly(anhydride-ester) (SAPAE) on the peri-implantitis progression in different systemic conditions based on reports that it reduces inflammation during bone regeneration.^63^ The null hypothesis postulated was that periodic local application of SAPAE would not influence peri-implantitis progression, irrespective of the systemic condition.

## 2 Materials and Methods

### 2.1 Implants

Titanium–zirconium alloy implants (3.3 × 8.0 mm) with progressive small buttress threads (thread pitch of 0.8 mm) possessing a sand-blasted/acid-etched surface (SLA, Bone Level, Straumann, Basel, Switzerland) were selected for the study. A total of 72 implants were utilized and randomly divided into 3 groups according to the systemic condition.

### 2.2 Preclinical *In Vivo* Model

This study was performed in accordance with the ethical approval from the Institutional Animal Care and Use Committee under institutional and national guidelines (protocol number #IA16-00195) as well as adhering to ARRIVE guidelines for reporting animal studies. Upon receiving approval, 18 female Göttingen minipigs (Marshal Laboratories, Clearwater, FL) with a minimum of 18 months of age were acquired and allowed to acclimate for 1 week prior to any surgical intervention.

### 2.3 Establishment of Metabolic Syndrome (MS) and Type-2 Diabetes Mellitus (T2DM) in Göttingen Minipig Models

Minipigs were randomly distributed into 3 groups using software, as follows: (i) control (normal diet), (ii) obesity/metabolic syndrome (MS) (cafeteria diet), and (iii) type-2 diabetes mellitus (T2DM) (cafeteria diet + streptozotocin) (*n* = 6/group). While a low-fat normal diet was provided to the control group animals, the metabolically impaired systemic condition (MS and T2DM) animals were fed a high-saturated and hydrogenated fats/cholesterol/sugar diet, a “cafeteria diet.”^64^ The animals were fed by the veterinarians twice a day with either a Standard Diet (SDS Standard Diet Service, UK #801586) or an RDS Cafeteria Diet (Research Diet Services NL), all with the same amount of food by weight.

First, to induce metabolic syndrome, 12 minipigs (both MS and T2DM groups) were steadily introduced to the cafeteria diet over a period of 4 weeks, with a weekly decrease of 25% in the normal diet, which is known as a conversion phase. During this phase, animals’ feeding was restricted to two 500 g meals per day. Thereafter, they remained at 100% cafeteria diet for 8 months, growth phase, and were fed *ad libitum*. Once MS and T2DM animals reached the desired body weight (approximately 50% increase relative to their original weight), the cafeteria diet was halved and combined with the control diet to maintain animals’ weight, maintenance phase. Control animals were fed a control diet and water throughout the experiment.

**Figure 1:**
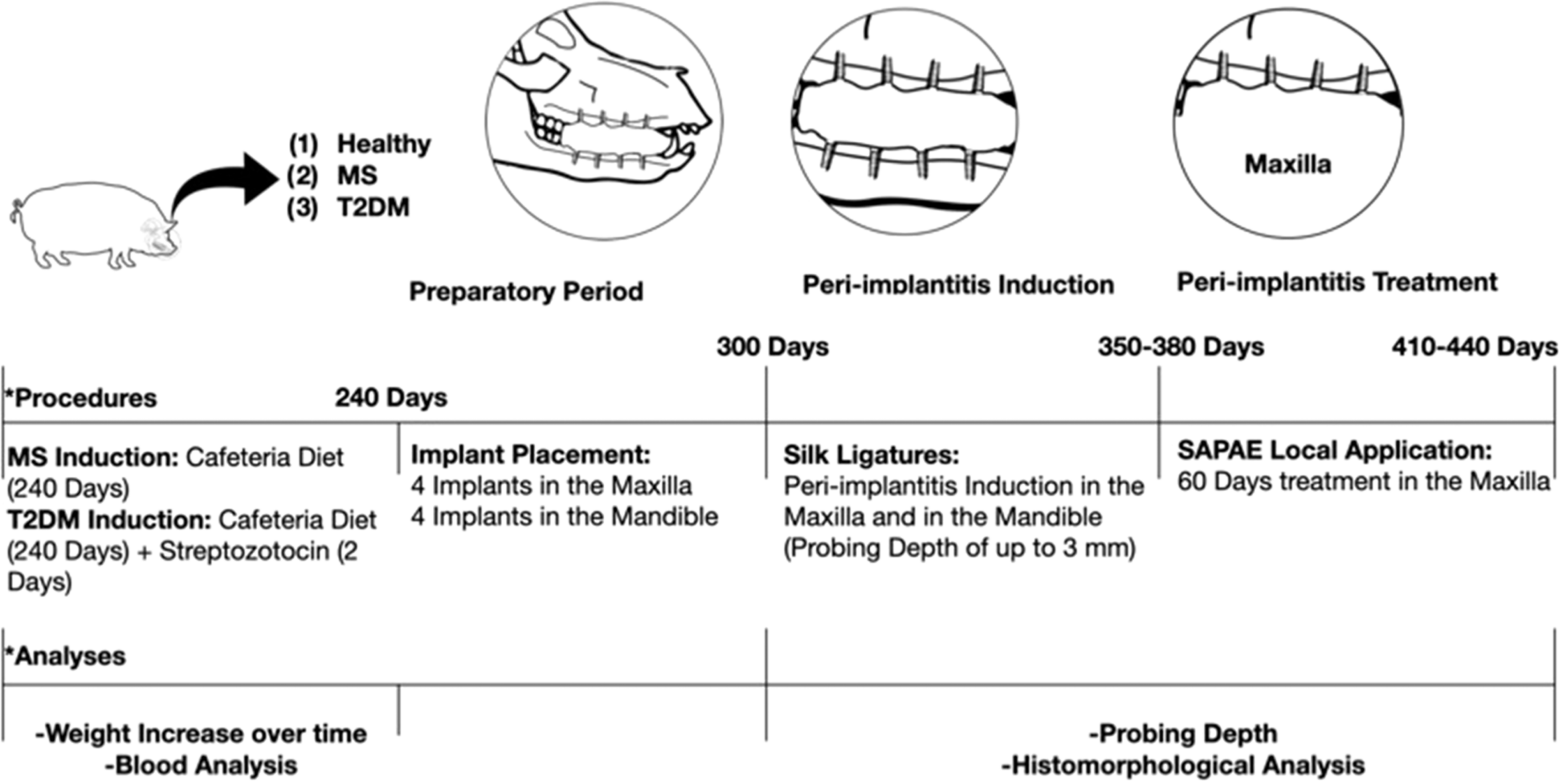
Flowchart of the study experiment design.

Second, to induce T2DM, six minipigs (T2DM group) were injected with a filter-sterilized β-cell cytotoxin streptozotocin solution (STZ, Enzo Life Sciences, Raamsdonksveer, The Netherlands) for two consecutive days (20 mg/kg in 0.1 mol/L Na-citrate, pH 4.5) following overnight fasting, as previously described.^65^ Free access to food following STZ injection was allowed during the daytime and after the second day of injection during the day and night. At the end of each of the first 2 days of STZ treatment, 25 g of glucose was fed to offset insulin release from β-cells, to prevent hypoglycemia (Figure 1). The induction of MS and T2DM onset through this proposed methodology was previously validated.^65^ In the present study, animals were monitored for characterization of metabolically compromised models relative to healthy controls, and no animals were excluded from the experiment. The following criteria were used to control the induction of MS and T2DM: animal weight and blood analysis (i.e., glucose, insulin, cholesterol, triglyceride, and cortisol levels).

### 2.4 Surgical Procedures

This study comprised of two surgical steps, which followed the same pre- and postsurgical protocol. Prior to any surgery, anesthesia was induced with ketamine hydrochloride 50 mg—Ketalar 50 mg/mL (20 mg/kg, Pfizer, New York, NY) and midazolam, Dormicum 5 mg/mL (Roche, Basel, Switzerland). ECG, SpO_2_, and end-tidal CO_2_ were used to monitor animals, and a circulating hot water blanket was utilized to maintain body temperature. In the first surgical procedure, maxillary and mandibular premolars and molar were extracted on the left side after being sectioned in the buccolingual direction. The soft tissue was closed with polypropylene 3-0 suture (Prolene, Ethicon, Johnson & Johnson, New Brunswick, NJ). The animals remained at the animal care facility and received antibiotic (Benzyl Penicillin Benzatine 20,000 UI/kg) and anti-inflammatory (Ketoprofen 1% 1 mL/5 kg) medication to control the pain and infection. After recovery, food and water *ad libitum* were offered to the animals by the responsible veterinarian. The suture was removed after 10 days, and the surgical site was observed to evaluate healing.

After 3 months, implants were placed in the left side, both in the mandible and in the maxilla. Full-thickness mucoperiosteal flaps were raised, the ridge was flattened under copious irrigation with sterile saline, and osteotomies were prepared according to the manufacturer’s recommendations. The implant osteotomy followed the drill sequence recommended by the manufacturer under abundant sterile saline irrigation at 1100 rpm. Once the implants were placed, closure caps were screwed and the soft tissue was closed. All postoperative procedures followed the abovementioned protocol. The implants were left to heal submerged for 2 months.

### 2.5 Peri-Implantitis Induction

**Figure 2:**
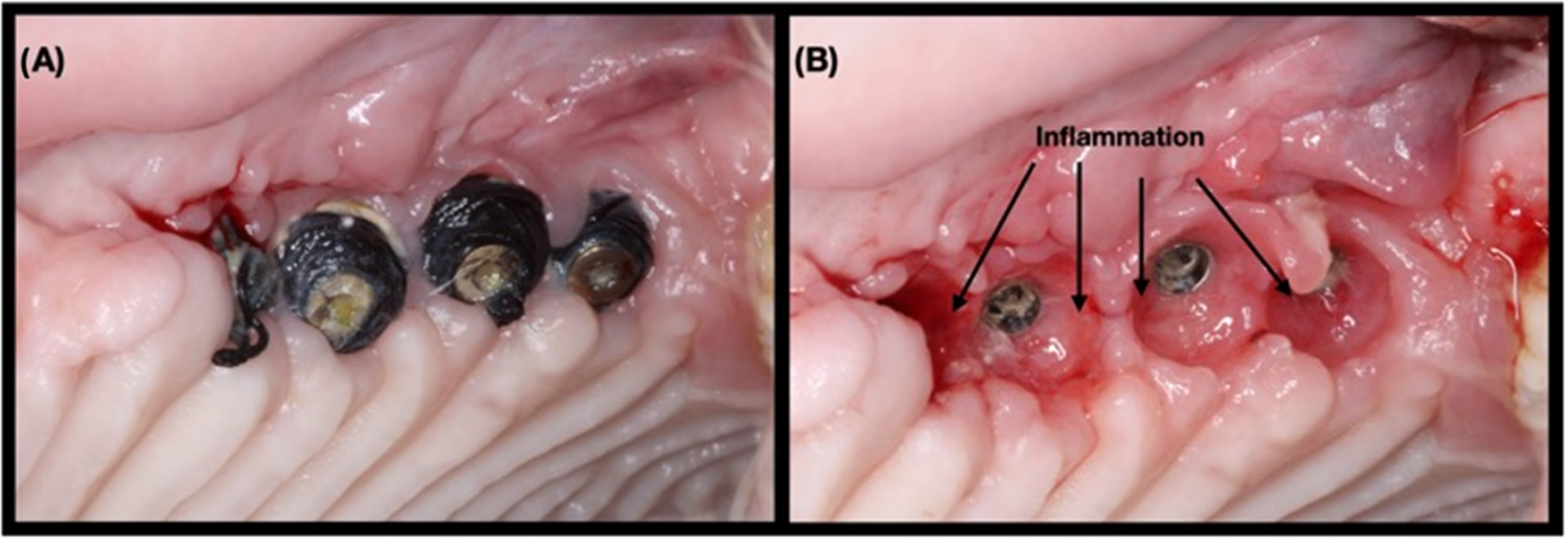
(A) Clinical aspect after 7 days of peri-implantitis induction using silk ligature, (B) where the presence of soft tissue inflammation can be observed.

After the healing period, implants were surgically uncovered, and closure caps removed and replaced with healing abutments. Again, all operative and postoperative procedures followed the aforementioned protocol, and healing occurred uneventfully. After 2 weeks of healing, oral hygiene procedures were purposefully neglected, and ligature-induced peri-implantitis was initiated. Silk ligatures were placed submarginally around the abutments to facilitate plaque accumulation and to induce plaque-associated peri-implant inflammation (Figure 2). Animals were sedated every 10 (±3) days with the same protocol mentioned above to examine ligatures and collect probing depth measurements of all implants by a single, trained operator. Peri-implantitis progression was examined for the different systemic conditions in the maxilla and mandible of the animals up to a total probing depth of approximately 3.0 mm, which would correspond to approximately 40% of bone loss relative to the total implant length (8.0 mm), compatible with values reported in previous studies investigating peri-implantitis induction using animal models.^66^

### 2.6 Peri-Implantitis Local Treatment

To halt peri-implantitis progression at failing implant sites, a local treatment was proposed with the local application of an anabolic agent, salicylic acid poly(anhydride-ester) (SAPAE). SAPAE polymer was synthesized by chemically incorporating salicylic acid (SA) in a poly(anhydride) using previously reported methods.^67^ In brief, the polymer precursor was synthesized in three steps where the carboxylic acid of salicylic acid was converted to a benzyl ester to give benzyl salicylate, with a free phenol group for further reaction with sebacoyl chloride to form bi(toluil-*o*-carboxyphenyl) adipate-polyethylene glycol. The benzyl groups were reductively cleaved to give the desired diacid, the monomer precursor. To form a polymer, the diacid was first acetylated with acetic anhydride to form the monomer, a mixed anhydride, which then underwent a melt condensation polymerization.^67^ The polymer was ground into a fine powder and mixed with mineral oil (3.75 mg/μL). The mixture was sterilized under ultraviolet light at 254 nm and 5500–6500 μW/cm^2^ for 15 min. SAPAE application as an ointment on the mucosa was initiated when the bone loss was approximately 1.5 mm, irrespective of systemic condition, in the maxilla. Implants were allocated into either control or SAPAE treatment groups in an interpolated distribution to minimize bias of the implantation site. The therapy protocol and probing depth assessment initially consisted of a weekly SAPAE local application on peri-implant sulcus and probing all implant surfaces, which was ineffective in slowing the progress of peri-implantitis, thus the regimen was changed to a biweekly local application. Peri-implantitis progression was followed for up to 60 days by probing around all quadrants of the implants every 10 (±3) days, independent of the systemic condition. Probing depth data were organized as a function of time. The animals were sacrificed by anesthesia overdose, and the samples were retrieved by sharp dissection.

### 2.7 Histologic Preparation and Histomorphologic Analysis

The samples were stored in 70% ethanol for 24 h and subjected to a progressive dehydration through a series of alcohol solutions ranging from 70 to 100% ethanol. Then, samples were embedded in a methacrylate-based resin according to the manufacturer’s instructions (Technovit 9100, Heraeus Kulzer GmbH, Wehrheim, Germany). The resin blocks were sectioned along with the implant long axis in a mesial-distal direction with a precision diamond saw (Isomet 2000, Buehler, Lake Bluff, IL) into ∼300 μm thick slices that were glued to acrylic plates with acrylate-based cement. After allowing 24 h for the samples to set and then were prepared for histological/metric analysis by grinding: 400–2400 grit SiC abrasive papers, and polishing: diamond suspension solutions of 1–9 μm particle size using a grinding/polishing machine (Metaserv 3000, Buehler, Lake Bluff, IL) under water irrigation until a final thickness of ∼100 μm. Thereafter, the samples were stained with Stevenel’s Blue and Van Giesons’s Picro Fuschin (SVG) stains and scanned via an automated slide scanning system and specialized computer software (Aperio Technologies, Vista, CA). Qualitative morphologic analysis was performed on the histologic images.

### 2.8 Statistical Analysis

Initial analyses of weight and blood, probing depth over time, and rate of probing depth increase per week yielded normal distribution (Shapiro-Wilk, all *p* > 0.05) and indistinguishable variances (Levene test, all *p* > 0.25). Weight and blood analysis data were statistically compared using analysis of variance and Tukey tests. Probing depth data were organized as a function of time (at every 10 days) and statistically evaluated through repeated measures analysis of variance following a posthoc comparison of the means using the Tukey test. Data of the rate of probing depth increase per week were also statistically evaluated through analysis of variance following pairwise comparisons using the Tukey test. Data are presented as mean and the corresponding 95% confidence interval (CI) values. All analyses were accomplished using SPSS (IBM SPSS 23, IBM Corp., Armonk, NY).

## 3 Results

**Figure 3:**
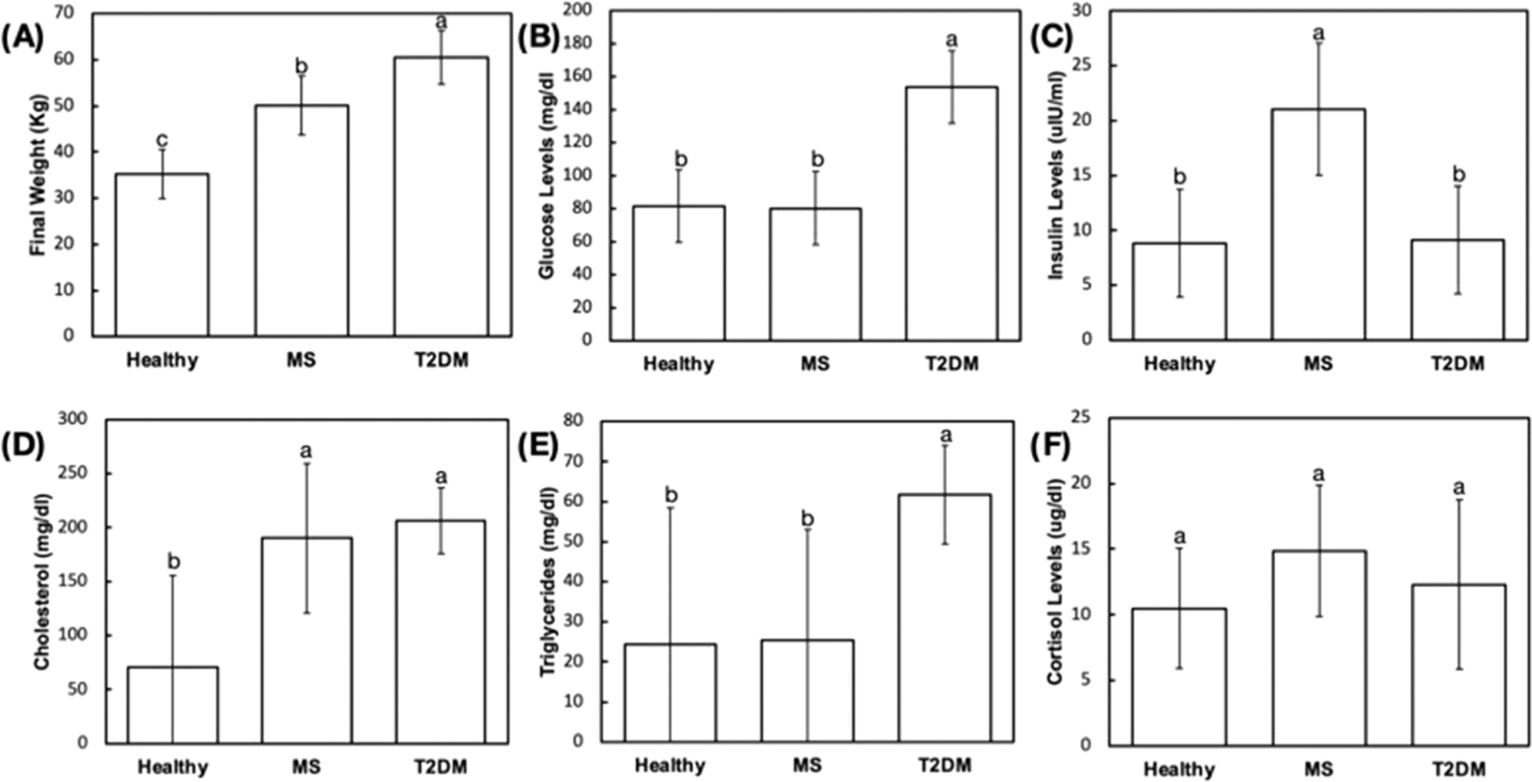
(A) Final weight and (B–F) blood marker profiles of the pigs to demonstrate the effective induction of a metabolically compromised condition. Different letters indicate statistically significant differences.

The final weight of pigs receiving the cafeteria diet and streptozotocin solution to induce type-2 diabetes mellitus (T2DM, 60.5 ± 5.7 kg) was significantly higher than of pigs receiving only the cafeteria diet to induce obesity/metabolic syndrome (MS, 50.1 ± 6.3 kg) and healthy pigs (35.2 ± 5.3 kg), respectively (*p* < 0.019) (Figure 3A). T2DM group presented average fasting blood glucose levels (153.8 ± 22 mg/dL) approximately 2-fold greater than MS (80.3 ± 22 mg/dL) and healthy (81.5 ± 22 mg/dL) groups (*p* < 0.001), without significant difference between the latter groups (*p* = 0.937) (Figure 3B). Plasma insulin levels were significantly greater in the MS group (21.0 ± 6 μLU/mL) relative to T2DM (9.1 ± 5 μLU/mL) and healthy groups (8.8 ± 5 μLU/mL) (*p* < 0.029), both without significant difference (*p* = 0.956) (Figure 3C). Pairwise comparisons of cholesterol levels of T2DM (206 ± 31 mg/dL) and MS (190 ± 69 mg/dL) groups were significantly higher than healthy control groups (70 ± 85 mg/dL) (*p* < 0.033) (Figure 3D). Triglyceride levels of T2DM subjects (61.8 ± 12.3 mg/dL) were greater than those observed for MS (25.3 ± 27 mg/dL) and healthy (24.3 ± 34 mg/dL) subjects (*p* < 0.043), both without significant difference (*p* = 0.964) (Figure 3E). Similarly, cortisol levels of metabolically compromised groups were slightly higher relative to the healthy control group, though no statistically significant difference was observed (*p* > 0.681) (Figure 3F).

**Figure 4:**
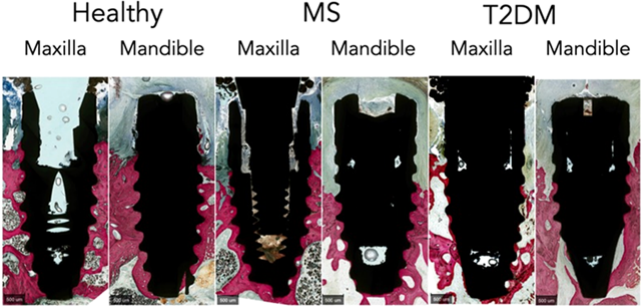
Histological micrographs of pigs with different systemic conditions demonstrating the effective induction of peri-implantitis with the proposed protocol in the maxilla and the mandible.

**Figure 5:**
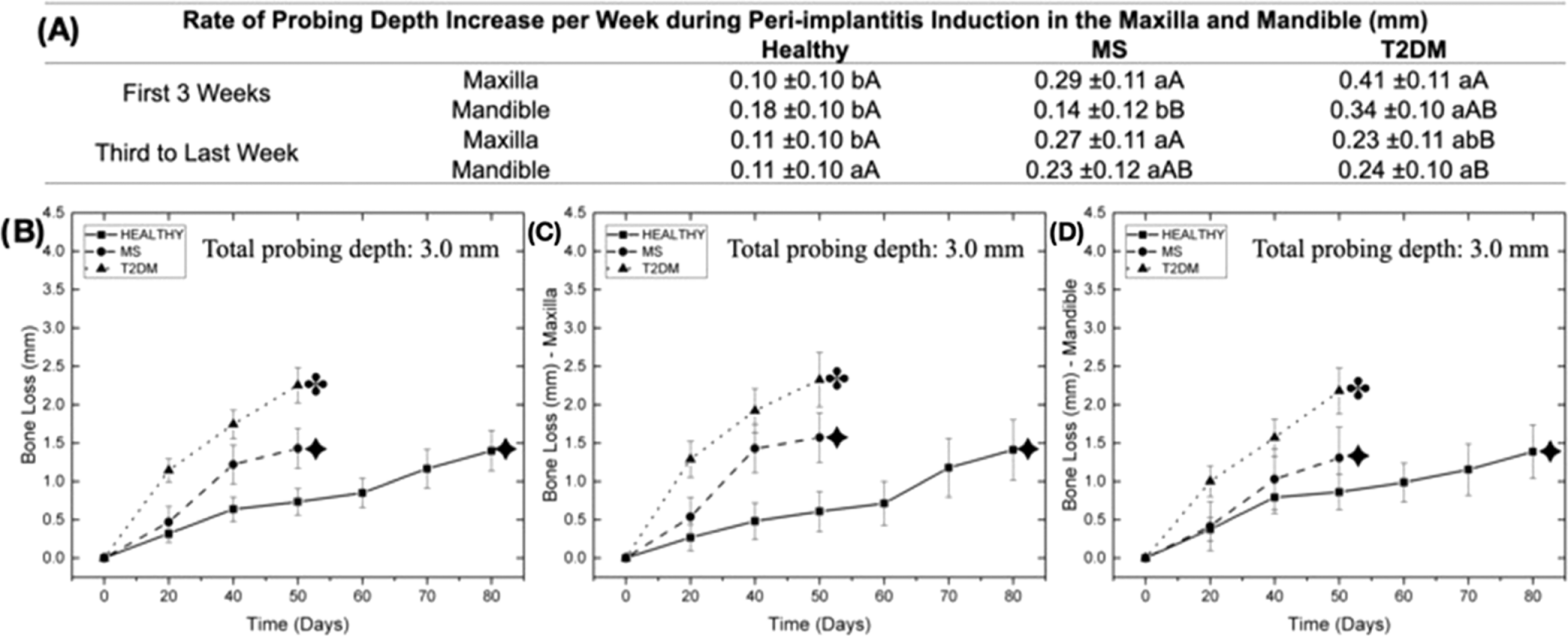
(A) Rate of probing depth increase per week for all systemic conditions and jaw region as a function of mean and 95% CI. Different lowercase letters indicate statistically significant differences between systemic conditions; different uppercase letters indicate statistically significant differences between time points. (B) Data collapsed over regions representing the probing depth after peri-implantitis induction using silk ligatures as a function of systemic condition. (C) Probing depth in the maxilla after peri-implantitis induction using silk ligatures as a function of systemic condition. (D) Probing depth in the mandible after peri-implantitis induction using silk ligatures as a function of systemic condition. Different symbols in the images (B), (C), and (D) indicate statistically significant differences in the bone loss values between systemic conditions at the end of the peri-implantitis induction.

Clinical evaluation of implants showed that plaque accumulation was associated with hyperplasia and redness of the mucosa surrounding the abutment ligatures. The marginal alveolar bone loss was confirmed in the histological micrographs, where tissue breakdown and more apical inflammatory progression, with approximately 3 mm probing depth from the implant shoulder, were observed for all images either in the maxilla or in the mandible, irrespective of systemic condition (Figure 4). Changes in attachment level were observed around the implants at different time points after peri-implantitis induction (Figure 5). Ligature placement resulted in an increase in the probing depth, with a more aggressive peri-implantitis progression in MS and T2DM relative to healthy control for both jaws, where healthy pigs (80 days) took approximately twice as long as metabolically compromised subjects (50 days) to reach similar levels of attachment loss (approximately 3 mm from the implant shoulder). After the initial 50 days of peri-implantitis induction using ligatures, MS and T2DM demonstrated approximately 50 and 65% more attachment loss relative to healthy subjects (*p* < 0.010). The rate of probing depth increase per week was significantly higher for implants placed in metabolically compromised animals relative to healthy animals, especially when data from T2DM subjects were compared with healthy subjects (*p* < 0.021). No statistically significant differences were detected with respect to attachment loss for maxilla and mandible pairwise comparisons, irrespective of systemic conditions (*p* > 0.086). Similarly, considering the rate of probing depth increase per week, no significant difference was observed for implants placed in the maxilla and the mandible for all systemic conditions (*p* > 0.184), except for MS pairwise comparison in the maxilla (*p* = 0.026) (Figure 5).

**Figure 6:**
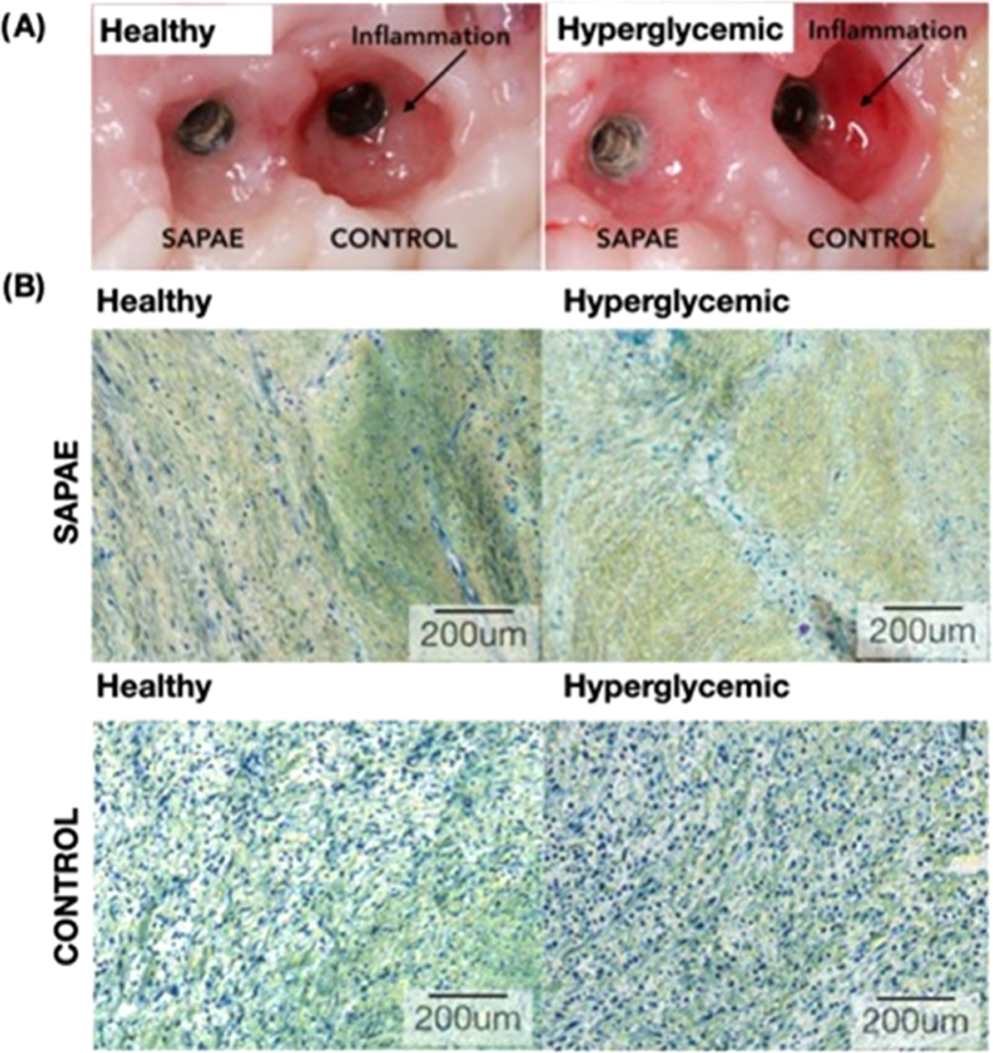
(A) Clinical aspect after 60 days of peri-implantitis halting treatment, where control implants presented substantial soft tissue inflammation. (B) High magnification of gingival connective tissue in proximity with the peri-implantitis-affected implants, where SAPAE-treated implants presented morphologic features of healthy peri-implant tissues in normoglycemic conditions and a slight elevation in the inflammatory content relative to healthy peri-implant issue for hyperglycemic conditions. Untreated control implants presented a substantially higher presence of inflammatory infiltrate relative to SAPAE-treated implants for both healthy and hyperglycemic groups.

As previously reported, plaque formation during experimental peri-implantitis induction resulted in evident signs of inflammation in the peri-implant mucosa for all implants; however, sites that underwent SAPAE therapy demonstrated a substantial decrease in the clinical signs of inflammation such as reduced redness, swelling, and bleeding on probing, even for the pro-inflammatory metabolically compromised condition, MS and T2DM (Figure 6A). High magnification micrographs of gingival connective tissue in proximity with the peri-implantitis-affected implants, where SAPAE-treated implants presented morphologic features of healthy peri-implant tissues in normoglycemic conditions and a slight elevation in inflammatory content relative to healthy peri-implant for hyperglycemic conditions. Untreated control implants presented a substantially higher presence of inflammatory infiltrate relative to SAPAE-treated implants for both healthy and more pronounced hyperglycemic groups (Figure 6B).

**Figure 7:**
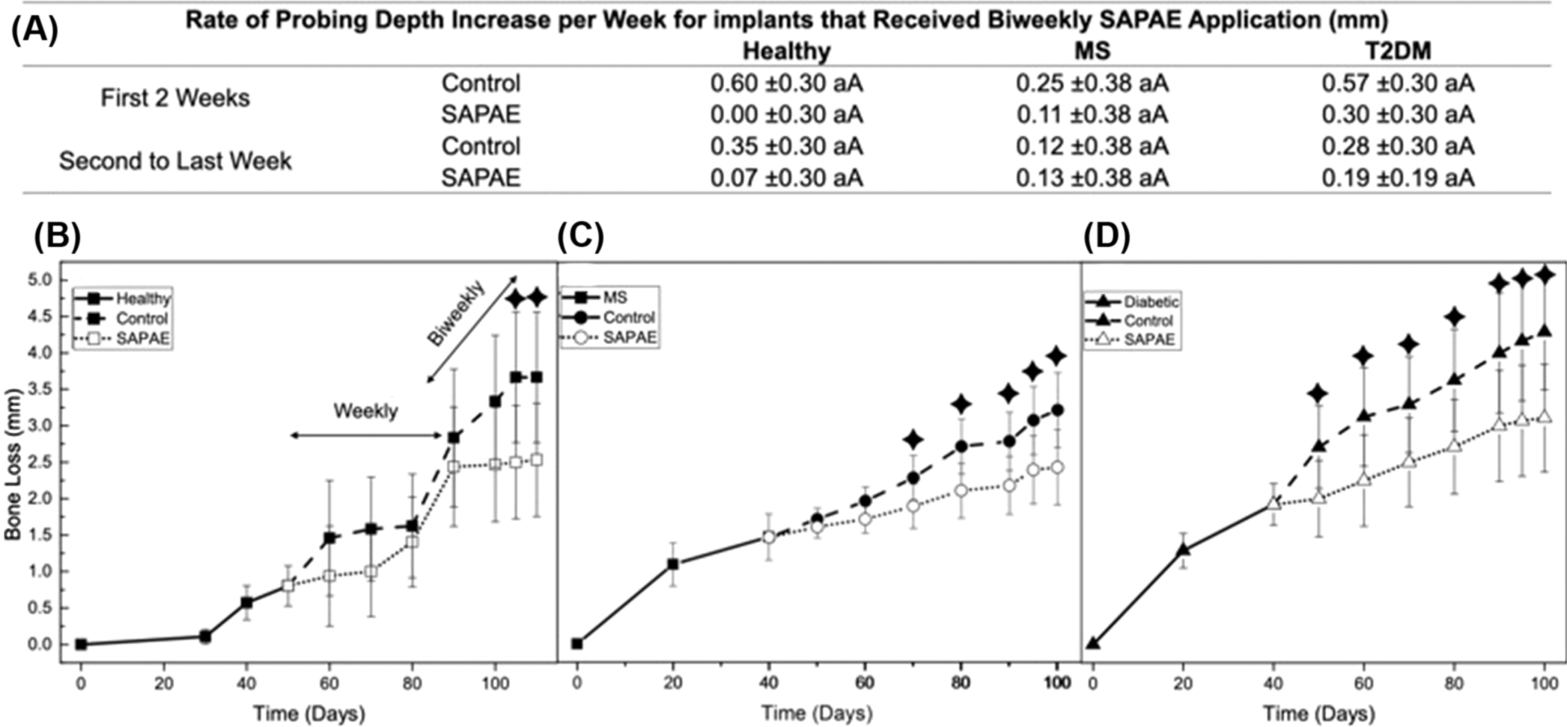
(A) Rate of probing depth increase per week for control and SAPAE groups as a function of mean and 95% CI. Different lowercase letters indicate statistically significant differences between systemic conditions; different uppercase letters indicate statistically significant differences between groups. Probing depth in the maxilla after peri-implantitis induction (approximately 1.5 mm) and the effect of SAPAE treatment in its progression as a function of systemic condition: healthy (B), MS (C), and T2DM (D). The symbol indicates time points with statistically significant differences between SAPAE and control groups. Therapy protocol was investigated in the healthy group, initially by a weekly SAPAE local application on peri-implant sulcus, which was ineffective in slowing the progress of peri-implantitis, thus the regimen was changed to a biweekly local application that successfully arrested peri-implantitis progression as a statistically significant lower probing depth can be observed for SAPAE group after approximately 15 days.

SAPAE therapy protocol was investigated in the healthy animals initially through a weekly local application of the drug on peri-implant sulcus; however, such a protocol was ineffective in slowing the progress of peri-implantitis, as observed in Figure 7 where no significant difference was observed in the probing depth between control and SAPAE groups (*p* > 0.186). Similarly, no significant difference was observed in the rate of probing depth increase per week during the period of weekly SAPAE application, approximately 30 days of treatment (control: 0.32 mm/week; SAPAE: 0.15 mm/week; *p* = 0.191). The regimen was then changed to a biweekly local application that successfully reduced peri-implantitis progression as SAPAE-treated implants presented substantially lower levels of bone loss relative to control groups, which was statistically significant after approximately 15 days of biweekly therapy for healthy pigs with almost 30% reduction in the probing depth relative to nontreated implants (*p* < 0.033). MS and T2DM presented a more distinct effect of SAPAE application on halting peri-implantitis progression, with significant differences between control and SAPAE groups after approximately 10 days of local treatment (*p* < 0.035). Similarly, an almost 30% reduction in the probing depth was observed for SAPAE-treated implants relative to nontreated implants (Figure 7). Despite differences in the attachment level, the rate of probing depth increase per week indicated no significant difference between the control and SAPAE groups for all pairwise comparisons (*p* > 0.196), except for healthy control animals in the first 2 weeks of treatment (*p* = 0.006). Similarly, systemic conditions showed no significant influence on the rate of probing depth increase per week (*p* > 0.155) (Figure 7).

## 4 Discussion

Obesity/metabolic syndrome (MS) is a risk factor for type-2 diabetes mellitus (T2DM) since over 90% of individuals with T2DM are obese.^68^ Currently, more than one-third of the adult population suffers from either MS or T2DM, and the prevalence is projected to steadily increase through 2050.^68,69^ Among the potential adverse consequences, the pro-inflammatory environment created by metabolic diseases because of persistent hyperglycemia and alterations in the host metabolism and potential consequence due to an altered oral microbiome is thought to be responsible for an early onset and more severe progression of peri-implantitis.^34−37^ Treatments for halting peri-implantitis progression have focused on implant surface decontamination along with adjunct therapies (i.e., antibiotics), which have yielded unpredictable results often with minimal improvement,^46,47,50,51,70,71^ especially in individuals with pro-inflammatory systemic conditions.^53−55^ The current study investigated the effect of periodic local application of salicylic acid poly(anhydride-ester) (SAPAE) and its effect on peri-implantitis progression in normal subjects or those with MS or T2DM. Implants that underwent periodic local SAPAE treatment in the maxilla demonstrated a substantial decrease in the clinical signs of inflammation, with a significant reduction in the probing depth relative to the control nontreated implants (approximately 30% reduction). Therefore, the postulated null hypothesis that periodic local application of SAPAE would not influence peri-implantitis progression, irrespective of systemic conditions, was rejected.

Current literature reporting preclinical experimental data regarding peri-implantitis pathogenesis and therapy usually employ large animal models, such as dogs, swine, and hon-human primates, due to similar anatomy, use of standard-size implants, and easier disease and/or treatment monitoring during the investigation, as well as analogous bone composition and metabolism relative to humans.^72,73^ As a shortcoming, large animal models present increased cost, longer healing time and experimental period, and necessity of appropriate equipment and facilities.^74^ Previous studies have validated the induction of MS and T2DM conditions in a Gottingen minipig model.^65,75,76^ In addition, T2DM onset has shown to be induced after MS development by a low dosage of streptozotocin, resembling the human metabolic compromise and pathophysiologic progression from MS to T2DM,^65,76,77^ with the animals presenting hyperglycemia, hyperlipidemia, hypertension, insulin resistance, and systemic inflammation.^65,78−82^ From a systemic compromise standpoint, the data obtained in the present study revealed that MS and T2DM Gottingen minipigs expressed similar disease phenotypes and progression, with high weight gain when subjected to a diet with high-saturated and hydrogenated fats and sugars (healthy: 35 kg; MS: 50 kg; and T2DM: 60 kg), insulin resistance, and elevated levels of cholesterol and triglycerides, as well as damage to β cells after the administration of streptozotocin in T2DM subjects, where the animals presented higher levels of hyperglycemia and elevated plasma cortisol.^65^ Additionally, a recent study has shown that both the MS and the T2DM Gottingen minipigs exhibited some level of impaired bone healing.^65^

The results show the successful induction, onset, and development of peri-implantitis using silk ligatures in both maxilla and mandible arches for either health or metabolically compromised Gottingen minipigs. The vast majority of preclinical animal studies use cotton or silk ligatures to induce peri-implantitis.^66^ Particularly, the configuration and size of peri-implant bone defects induced by ligatures (herein usually circumferential bone defects without dehiscence, as observed in the histologic micrographs) as well as the associated microflora have shown to closely resemble human conditions.^66,83^ Previous microbiological analyses have revealed an increased level of Gram-negative anaerobic bacteria, such as *Porphyromonas gingivalis*, *Prevotella intermedia*, and *Tannerella forsythia* and, occasionally, *Campylobacter spp*. and *Candida spp*. in ligature-induced peri-implantitis in different animal models, including swine,^66,84^ which were also common to peri-implant infections in humans that are mainly characterized by high counts of *P. gingivalis*, *P. intermedia*, *T. forsythia*, *Treponema denticola*, *Aggregatibacter actinomycetemcomitans*, among others.^85−87^

From a temporal perspective, the progression of the disease was faster in MS and T2DM subjects in both the mandible and the maxilla relative to normoglycemic healthy subjects. This may be accounted for by the peri-implant crevicular fluid of patients with peri-implantitis exhibiting increased inflammation with higher levels of interleukin (IL) 1, IL-8, and tumor necrosis factor (TNF) α, as well as matrix metalloproteinase collagenase 8 relative to the crevicular fluid of healthy patients.^88,89^ Increased expression of IL-8 and TNF-α has also been observed in the peri-implant crevicular fluid of individuals with poor glycemic control compared with well-controlled glucose levels and healthy individuals.^55,88^

Current methods applied for the treatment of peri-implantitis have focused on implant surface decontamination (with approximately 0.23 mm reduction in the probing depth after treatment^71^), local and systemic administration of antibiotics (∼0.27–0.30 mm reduction in the probing depth^70,71^), as well as regenerative procedures using different types of bone grafts and membranes (∼0.51 mm reduction in the probing depth).^48,49,71^ The results obtained have indicated minimal bone attachment gain and probing depth reduction,^45,48^ especially in individuals with pro-inflammatory systemic conditions.^48,53−56^ In the current study, the local periodic application of salicylic acid in an attempt to overcome the exacerbated inflammatory response of peri-implant diseases demonstrated a substantial decrease in the clinical signs of inflammation, such as redness, swelling, and bleeding on probing, even under pro-inflammatory MS and T2DM conditions. Untreated control implants presented a substantially higher presence of inflammatory infiltrate relative to SAPAE-treated implants in the histologic micrographs for healthy and more pronounced hyperglycemic groups. Furthermore, the application of SAPAE around implants halted peri-implantitis progression, as treated groups presented significantly lower levels of probing depth relative to untreated groups after approximately 10–15 days of therapy for healthy and metabolically compromised minipigs, with almost 30% reduction in the probing depth. The rationale behind the positive result lies in the sustained local release of salicylic acid from SAPAE that inhibits inflammatory pathways.^63,90−94^ Salicylic acid has been shown to decrease the activation of nuclear factor kappa-light-chain-enhancer of activated B cells (NF-κB), reducing the production of pro-inflammatory cytokines (IL and TNF-α), that impair bone metabolism and enhance bone resorption, as mentioned above, thus providing a more favorable scenario for bone regeneration.^63,95^ Also, the more evident effect of SAPAE on metabolically compromised conditions, MS and T2DM, might be potentially associated with the chronic disease-enhanced inflammatory state, where the salicylic acid anti-inflammatory effect could have led to a greater difference between the experimental and control groups.^63^

Biodegradable and bioabsorbable polymeric carriers designed for drug transporters, such as salicylic acid poly(anhydride-ester) (SAPAE), have emerged as safe and efficient therapies that control the rate, time, and place of drug release, being amenable to various formulations for drug delivery and handling.^91,93,94,96−98^ The local and sustained release of drugs may represent a breakthrough for increasing the predictability of peri-implantitis treatment, as demonstrated by the current results, encouraging future investigations to evaluate the benefits of using different dosages and/or protocols as well as different types of drugs and/or drug combinations. A previous study has also demonstrated the positive effects of adding SAPAE powder to grafted areas in small animal models for either healthy or hyperglycemic systemic conditions,^63^ which along with the current data encourages future highly translational studies to investigate the benefits of associating local and sustained release of anti-inflammatory drugs in regenerative procedures.^63^

## 5 Conclusions

Metabolically compromised conditions, obesity/metabolic syndrome (MS), and type-2 diabetes mellitus (T2DM) were successfully induced in the Gottingen minipig model. The silk ligature-induced protocol resulted in the onset and development of peri-implantitis, irrespective of systemic conditions; though a faster progression was observed for MS and T2DM. Also, the local and sustained release of salicylic acid halted the progression of peri-implantitis in either healthy or metabolically compromised systemic conditions.
